# Serum proteomic profiling reveals fragments of MYOM3 as potential biomarkers for monitoring the outcome of therapeutic interventions in muscular dystrophies

**DOI:** 10.1093/hmg/ddv214

**Published:** 2015-06-09

**Authors:** Jérémy Rouillon, Jérôme Poupiot, Aleksandar Zocevic, Fatima Amor, Thibaut Léger, Camille Garcia, Jean-Michel Camadro, Brenda Wong, Robin Pinilla, Jérémie Cosette, Anna M.L. Coenen-Stass, Graham Mcclorey, Thomas C. Roberts, Matthew J.A. Wood, Laurent Servais, Bjarne Udd, Thomas Voit, Isabelle Richard, Fedor Svinartchouk

**Affiliations:** 1 Généthon, Evry, France,; 2 Mass spectrometry Laboratory, Institut Jacques Monod, UMR 7592, University Paris Diderot, CNRS, Sorbonne Paris Cité, F-75205 Paris, France,; 3 Division of Pediatric Neurology, Cincinnati Children's Hospital Medical Center, Cincinnati, OH, USA,; 4 Department of Physiology, Anatomy and Genetics Oxford, Oxford, OX1 3QX, UK,; 5 Department of Molecular and Experimental Medicine, The Scripps Research Institute, La Jolla, CA, USA,; 6 Service of Clinical Trials and Databases, Institut de Myologie, Paris, France,; 7 Folkhälsan Institute of Genetics and Department of Medical Genetics, Haartman Institute, University of Helsinki, Helsinki, Finland,; 8 UPMC Inserm, UMRS 974, CNRS FRE 3617, Paris, France,; 9 Université Pierre et Marie Curie- Paris 6, Institut de Myologie, GH Pitié-Salpêtrière, Paris, France and; 10 Genethon, CNRS UMR 8587, Evry, France

## Abstract

Therapy-responsive biomarkers are an important and unmet need in the muscular dystrophy field where new treatments are currently in clinical trials. By using a comprehensive high-resolution mass spectrometry approach and western blot validation, we found that two fragments of the myofibrillar structural protein myomesin-3 (MYOM3) are abnormally present in sera of Duchenne muscular dystrophy (DMD) patients, limb-girdle muscular dystrophy type 2D (LGMD2D) and their respective animal models. Levels of MYOM3 fragments were assayed in therapeutic model systems: (1) restoration of dystrophin expression by antisense oligonucleotide-mediated exon-skipping in *mdx* mice and (2) stable restoration of α-sarcoglycan expression in KO-SGCA mice by systemic injection of a viral vector. Following administration of the therapeutic agents MYOM3 was restored toward wild-type levels. In the LGMD model, where different doses of vector were used, MYOM3 restoration was dose-dependent. MYOM3 fragments showed lower inter-individual variability compared with the commonly used creatine kinase assay, and correlated better with the restoration of the dystrophin-associated protein complex and muscle force. These data suggest that the MYOM3 fragments hold promise for minimally invasive assessment of experimental therapies for DMD and other neuromuscular disorders.

## Introduction

The dystrophin-associated protein complex (DAPC) consists of several transmembrane and intracellular scaffolding elements implicated in maintaining the structure and morphology of vertebrate muscle fibres. Loss-of-function mutations in genes encoding these proteins give rise to different forms of muscular dystrophy. The absence of functional dystrophin or sarcoglycans in the DAPC is accompanied by a strong destabilization of the complex at the sarcolemma (1). As a consequence, muscle fibres become more sensitive to mechanical damage leading to muscle degeneration, chronic inflammation and an increase in fibrosis—hallmarks of the dystrophic phenotype (2). The most prevalent and severe disease is Duchenne muscular dystrophy (DMD), an X-linked disorder caused by mutations in the dystrophin gene, with a world-wide incidence of 1/5000 male newborns. DMD patients usually lose the ability to walk around the age of 12 and die in their third or fourth decade due to cardiorespiratory complications (3). Deficiencies in the sarcoglycan genes are usually less severe but can also be accompanied by cardiac problems (4,5).

Recently, substantial progress in the development of therapeutic approaches for the treatment of muscular dystrophies has been accomplished. Therapies for DMD based on the delivery of minidystrophin (6) or antisense oligonucleotide-mediated exon-skipping (7–10) are in pre-clinical evaluation or in phase I–III clinical trials. The small-molecule compound Ataluren (11,12) has recently obtained conditional marketing authorization for the treatment of DMD. Furthermore, a long-term, sustained restoration of α-sarcoglycan (Sgca) and γ-sarcoglycan (Sgcg) expression was observed following intramuscular gene transfer to muscles of patients with limb-girdle muscular dystrophy types 2D (LGMD2D) (13) and 2C (14) respectively. With recent progress in pharmaco- or gene-therapy for muscular dystrophies there is a growing need for minimally invasive biomarkers that can be used to assess and monitor the efficacy of therapy. Indeed, in order to evaluate the efficiency of a treatment during animal studies, researchers have unlimited access to different types of biopsies or necropsies. In contrast, trials in humans impose ethical restrictions requiring minimally invasive methods to assess and monitor the efficacy of therapy. Current methods include functional evaluation scales to measure patients’ status (15–17), measurement of the level of fatty infiltration by magnetic resonance imaging (MRI) (18) and quantification of serum microRNAs (19–21) or urinary proteins (22). The biomarker most commonly used for DMD is serum creatine kinase (CK), which leaks into the blood stream upon muscle damage. However, CK demonstrates variations due to physical activity, muscle injury, cramping, toxic agents or age (23,24). Thus, although serum CK measurement is a useful diagnostic biomarker (25), it is not appropriate to predict the course of disease, severity of pathology or to monitor the efficacy of treatment.

Variations in the composition of serum proteome are considered a promising source of biomarkers (26). In the present study, serum samples from DMD patients and healthy controls were compared using a comprehensive high-resolution mass spectrometry approach and several tens of proteins with altered levels were revealed by label-free protein quantification analysis. Among these proteins, the myofibrillar structural protein myomesin-3 (MYOM3), which was more abundant in DMD patient sera than in healthy controls, was chosen for detailed analysis. MYOM3 was present in sera as two internal fragments of 100 and 130 kDa rather than as an intact protein. Importantly, these fragments demonstrated lower inter-individual variations compared to CK. High levels of these MYOM3 fragments were also detected in sera from LGMD2D patients, as well as in animal models of DMD and several limb-girdle muscular dystrophies.

In the dystrophin-deficient *mdx* mouse, these fragments were more reliable for the early detection of the disease and less sensitive to physical exercise when compared to CK. MYOM3 fragments were also superior when compared to CK for the monitoring the restoration of the DAPC and correlated to the rescue of physical force after gene therapy treatment of LGMD2D mouse model. Taken together, our data suggest that MYOM3 fragments are biomarkers for the detection, evaluation and treatment monitoring of DMD, LGMD2D and potentially for other forms of muscular dystrophy associated with increased turnover of sarcomeric proteins.

## Results

### Detection of serum proteins with altered levels in DMD patients by mass spectrometry

Serum samples from 39 DMD patients and 38 control subjects collected in USA as part of the Advanced Diagnostics for New Therapeutic Approaches (ADNA) project (http://www.institut-merieux.com/projetssante_adna.php) were analysed using a mass spectrometry approach. To reduce the number of LC-MS/MS analyses, the samples were organized into four groups (G1: young DMD from 3 to 10 years old; G2: older DMD from 12 to 20 years old; G3: young controls from 3 to 10 years old and G4: older controls from 12 to 20 years old) subdivided in a total of 12 pools according to the patient's age (Table 1). Each pool included sera from at least four individuals where serum of each individual was equally represented. In order to ensure deep proteome coverage, the pools were immunodepleted for the 12 major serum proteins.

**Table 1.** Schema of samples assembling into groups and subgroups. **DMD G1-1 to G1-4**: serum from young DMD patients of 3 to 10 years old; **DMD G2-1 to G2-2**: serum from DMD patients of 12 to 20 years old. **Healthy controls G3-1 to G3-4** and **G4-1 to G4-2**: age-matched healthy controls to the young and older DMD patients respectively. Numbers below each pool indicate the interval of age and the number of patients (in brackets)

**DMD**	**G1–1**	**G1–2**	**G1–3**	**G1–4**	**G2–1**	**G2–2**
Age (number)	3–4 (7)	4–6 (11)	6–7 (4)	7–10 (4)	12–16 (6)	16–20 (7)
**Healthy controls**	**G3–1**	**G3–2**	**G3–3**	**G3–4**	**G4–1**	**G4–2**
Age (number)	3–4 (5)	4–6 (6)	6–7 (5)	7–10 (5)	12–16 (10)	16–20 (7)

Mass spectrometry analysis of serum samples of all 12 subgroups enabled the identification a total of 3329 unique peptides matching 378 proteins (with a false discovery rate less than 0.01). Among those, 69% of protein identification calls (260 proteins) were based on spectra from two or more peptides. To reveal proteins differentially present in sera from DMD and healthy individuals, the data were analysed by a label-free quantification approach using the following parameters: number of peptides ≥2; Mascot protein score (http://www.matrixscience.com/) ≥50 and fold change ≥2. The analysis of young DMD patients with their age-matched controls (G1 versus G3 groups) revealed 24 proteins more abundant in DMD and 13 in healthy subjects (Table 2). The top 10 proteins with the lowest *P*-value were more abundant in DMD patients and either involved in muscle energy metabolism (pyruvate kinase PKM, L-lactate dehydrogenase B chain, CK-M, alanine aminotransferase 1, β-enolase, carbonic anhydrase 3, fructose-bisphosphate aldolase A), in sarcomere organization (myomesin-3, myosin-7) or costamere organization (vinculin).

**Table 2.** List of proteins with altered levels between G1 and G3 groups (young DMD and age-matched healthy controls) classified by the decrease in the ratio DMD/healthy (fold change)

No. accession	Description	Localization	Peptides	Score	ANOVA (*P*-value)	Fold change
MYG_HUMAN	Myoglobin	Cytoplasm	4	195	2.7e-03	234.8
MYOM2_HUMAN	MYOM2	Myofibril	10	390	9.8e-05	100.1
**MYOM3_HUMAN**	**MYOM3**	**Myofibril**	**11**	**491**	**1**.**5e-05**	**49**.**7**
TPIS_HUMAN	Triosephosphate isomerase	Cytoplasm	3	128	2.3e-03	48.4
AATC_HUMAN	Aspartate aminotransferase	Cytoplasm	3	75	4.7e-04	45.7
**KCRM_HUMAN**	**CK-M**	**Cytoplasm**	**15**	**849**	**2**.**9e-05**	**39**.**8**
**MYH7_HUMAN**	**Myosin-7**	**Myofibril**	**11**	**520**	**2**.**2e-05**	**38**.**3**
**ENOB_HUMAN**	**β-enolase**	**Cytoplasm**	**4**	**178**	**7**.**4e-05**	**34**.**8**
G6PI_HUMAN	Glucose-6-phosphate isomerase	Cytoplasm/Secreted	4	130	1.6e-03	29.5
**CAH3_HUMAN**	**Carbonic anhydrase 3**	**Cytoplasm**	**5**	**182**	**8**.**6e-05**	**23**.**9**
FLNC_HUMAN	Filamin-C	Myofibril	4	145	4.3e-04	19.4
**ALAT1_HUMAN**	**Alanine aminotransferase 1**	**Cytoplasm**	**4**	**127**	**3**.**0e-05**	**15**.**6**
**ALDOA_HUMAN**	**Fructose-bisphosphate aldolase A**	**Cytoplasm**	**15**	**729**	**9**.**3e-05**	**14**.**2**
**KPYM_HUMAN**	**Pyruvate kinase PKM**	**Cytoplasm**	**16**	**845**	**1**.**1e-05**	**12**.**8**
TITIN_HUMAN	Titin	Myofibril	14	495	1.9e-03	10.8
**VINC_HUMAN**	**Vinculin**	**Cytoplasm/Membrane**	**2**	**74**	**7**.**2e-05**	**10**.**3**
PYGM_HUMAN	Glycogen phosphorylase, muscle form	Cytoplasm	8	257	6.1e-04	9.9
LDHA_HUMAN	L-lactate dehydrogenase A chain	Cytoplasm	8	378	9.1e-04	9.5
HPT_HUMAN	Haptoglobin	Secreted	29	1867	1.5e-04	7.6
HBD_HUMAN	Haemoglobin subunit δ	Cytoplasm	3	100	5.1e-03	6.2
**LDHB_HUMAN**	**L-lactate dehydrogenase B**	**Cytoplasm**	**10**	**598**	**2**.**4e-05**	**5**.**4**
HBB_HUMAN	Haemoglobin subunit β	Cytoplasm	7	552	8.0e-03	3.6
HBA_HUMAN	Haemoglobin subunit α	Cytoplasm	7	407	5.3e-03	3.4
TPM2_HUMAN	Tropomyosin βchain	Myofibril	5	170	2.0e-02	2.6
VASN_HUMAN	Vasorin	Membrane	4	135	4.0e-02	0.5
ALS_HUMAN	Insulin-like growth factor-binding protein complex	Secreted	22	1096	1.0e-02	0.5
PHLD_HUMAN	Phosphatidylinositol-glycan-specific phospholipase D	Secreted	9	533	4.7e-03	0.5
CHL1_HUMAN	Neural cell adhesion molecule L1-like protein	Membrane/Secreted	2	66	3.0e-02	0.5
COL11_HUMAN	Collectin-11	Secreted	2	72	2.6e-03	0.4
CADH5_HUMAN	Cadherin-5	Membrane	6	220	2.0e-03	0.4
CD109_HUMAN	CD109 antigen	Membrane	2	59	3.0e-02	0.4
LBP_HUMAN	Lipopolysaccharide-binding protein	Secreted	7	386	5.0e-03	0.4
CRAC1_HUMAN	Cartilage acidic protein 1	Secreted	6	223	2.0e-02	0.4
C4BPB_HUMAN	C4b-binding protein	Secreted	4	207	2.0e-02	0.4
CNDP1_HUMAN	β-Ala-His dipeptidase	Secreted	8	294	4.2e-03	0.3
DPP4_HUMAN	Dipeptidyl peptidase 4	Membrane/Secreted	5	162	5.5e-03	0.3
CETP_HUMAN	Cholesteryl ester transfer protein	Secreted	7	296	8.2e-04	0.2

Top 10 proteins with the lowest *P*-value are in bold. All shown proteins passed thresholds of peptide numbers ≥2, a score≥50, a fold change ≥ 2 and a *P*-value ≤ 0.05. Peptides: number of peptides identified for a given protein. Score: Mascot protein score.

Comparison of older DMD patients with their age-matched controls (G2 versus G4) using the same parameters resulted in only nine altered proteins: five proteins more abundant in DMD (CK-M, adiponectin, fructose-bisphosphate aldolase A, L-lactate dehydrogenase B chain and haemoglobin β) and four in healthy subjects (gelsolin, phosphatidylcholine-sterol acyltransferase, cadherin-13 and cartilage acidic protein 1) (Table 3). Only four of these proteins (CK-M, fructose-bisphosphate aldolase A, L-lactate dehydrogenase B chain and haemoglobin β) were differentially abundant in both DMD age groups according to the mass spectrometry analysis. Importantly, the expression ratios for these four proteins in DMD versus healthy controls were substantially lower in older DMD patients as compared to the young DMD group (19.5; 3.3; 2.2 and 2.4 folds in older DMD versus 39.8; 14.2; 5.4 and 3.6 times in young, respectively). The decrease in the number of proteins with altered levels and in magnitude of their fold changes is most probably due to the drastic decrease of muscle mass in older DMD patients and relative immobility of these patients. Interestingly, label-free analysis of young and older DMD patients (G1 versus G2) revealed eight secreted proteins that increased in abundance with patient age (dopamine β-hydroxylase: 3-fold, adiponectin: 3-fold, serum amyloid P-component: 3-fold, insulin-like growth factor-binding protein complex acid labile subunit: 3-fold, β-Ala-His dipeptidase: 5-fold, insulin-like growth factor I: 5-fold).

**Table 3.** List of proteins with altered levels in serum samples between G2 and G4 groups (older DMD and age-matched healthy controls) classified by the decrease in the ratio DMD/healthy (fold change)

No. accession	Description	Localization	peptides	Score	ANOVA (*P*-value)	Fold change
KCRM_HUMAN	CK-M	Cytoplasm	3	96	1.0e-02	19.5
ADIPO_HUMAN	Adiponectin	Secreted	3	213	3.0e-02	4.4
ALDOA_HUMAN	Fructose-bisphosphate aldolase A	Cytoplasm	2	84	3.0e-02	3.3
HBB_HUMAN	Haemoglobin subunit β	Cytoplasm	9	632	8.4e-03	2.4
LDHB_HUMAN	L-lactate dehydrogenase B chain	Cytoplasm	6	223	4.0e-02	2.2
GELS_HUMAN	Gelsolin	Cytoplasm	32	2287	1.0e-02	0.5
LCAT_HUMAN	Phosphatidylcholine-sterol acyltransferase	Secreted	6	295	1.0e-02	0.4
CAD13_HUMAN	Cadherin-13	Membrane	2	86	5.8e-03	0.4
CRAC1_HUMAN	Cartilage acidic protein 1	Secreted	3	105	1.0e-02	0.2

All shown proteins passed thresholds of peptide numbers ≥2, a score ≥ 50, a fold change ≥ 2 and a *P*-value ≤ 0.05. Peptides: number of peptides identified for a given protein. Score: Mascot protein score.

### Levels of MYOM3 demonstrate less inter-individual variations compared to CK in DMD patients

Elevated levels of cytosolic proteins such as CK in the blood are now widely used as the first stage of DMD diagnosis (24,27). Therefore, it was appealing to compare serum levels of CK with one of the myofibrillar structural proteins found in the present study. Serum biomarkers are often presented by truncated fragment of the full-length proteins, thus making difficult finding of commercial antibodies for the specific fragment (28). In our hands, of the eleven antibodies tested against the three myofibrillar structural proteins (MYOM2, MYOM3 and Myosin 7) with the highest high fold change and lowest *P*-value between DMD and healthy controls (Table 2) only antibody against MYOM3 was efficient in immunoblot analysis. MYOM3 (UniProtKB # Q5VTT5), a protein of 1437 amino acids (162.2 kDa), is a member of a family of closely related structural proteins detected at the M-band of the sarcomere in striated skeletal muscles: MYOM1, MYOM2 (or M protein) and MYOM3. These proteins are involved in sarcomere stability and resistance during intense or sustained stretching (29).

**Figure 1.**
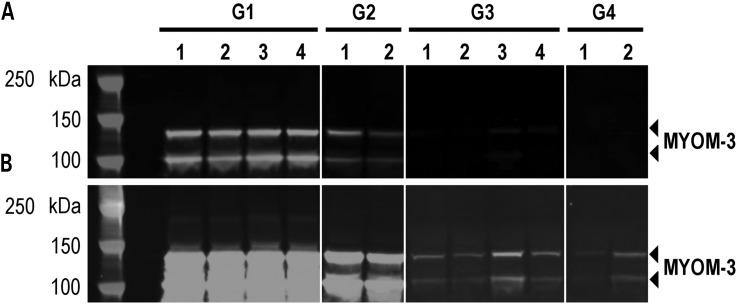
Western blot analysis of MYOM3 in pools of sera from subgroups of young (G1) and older (G2) DMD patients as well as young (G3) and older (G4) healthy subjects. (**A**) normal exposure; (**B**) boosted exposure. For explanation of groups and subgroups see Table 1. Fifty micrograms of serum proteins were loaded in each well.

Western blot analysis of serum from DMD patients with an anti-MYOM3 antibody targeting amino acids 887–1178 of the protein revealed the presence of two bands of 100 and 130 kDa respectively (Fig. 1). To identify the position of these fragments within the protein, they were purified by immunoprecipitation, separated by SDS-PAGE and analysed by mass spectrometry separately (Table 4). The obtained peptide coverages of the fragments (total 55 peptides covering amino acid 254–1331 for the upper fragment and 38 peptides covering the sequence from amino acid 476–1331 for the lower fragment) suggest that both fragments have similar C-terminal end but different N-terminus. Minimum molecular weight of the fragments based on the positions of the most N-terminal and C-terminal identified peptides is equal to 121 kDa for the upper fragment (1077 aa) and 96 kDa (855 aa) for the lower fragment, which fit well with the size of the fragments estimated by SDS-PAGE (130 and 100 kDa respectively). Importantly, fragments of the same size were barely detectable in sera from healthy subjects by Western blot analysis (Fig. 1), thus validating the mass spectrometry data.

**Table 4.** List of MYOM3 peptides identified by mass spectrometry in upper (∼130 kDa) and lower (∼100 kDa) bands from DMD patient serum

#	Position	Sequence	Ion score (Upper band)	Ion score (Lower band)
1	254–263	DAGFDSEIFK	24	
2	265–283	STFGPSVEFTSVLKPVFAR	44	
3	320–326	KILYTDR	19	
4	321–326	ILYTDR	12	
5	338–346	EDEGLYMVR	57	
6	347–354	VPSPFGPR	44	
7	355–363	EQSTYVLVR	48	
8	364–380	DAEAENPGAPGSPLNVR	54	
9	401–411	GNPITAYTIER	40	
10	461–474	ASELVVMGDHDAAR	54	
11	476–486	KTEIPFDLGNK	64	53
12	477–486	TEIPFDLGNK	19	43
13	487–513	ITISTDAFEDTVTIPSPPTNVHASEIR	92	50
14	514–527	EAYVVLAWEEPSPR	65	55
15	530–538	APLTYSLEK	66	37
16	539–556	SVIGSGTWEAISSESPVR	86	57
17	560–567	FAVLDLEK	40	37
18	560–568	FAVLDLEKK	33	35
19	569–574	KSYVFR	32	
20	577–596	AMNQYGLSDPSEPSEPIALR	69	58
21	597–612	GPPATLPPPAQVQAFR	79	42
22	613–638	DTQTSVSLTWDPVKDPELLGYYIYSR	33	18
23	639–657	KVGTSEWQTVNNKPIQGTR	27	23
24	640–657	VGTSEWQTVNNKPIQGTR	67	79
25	658–664	FTVPGLR	25	21
26	675–692	SVSEAGVGESSAATEPIR	96	63
27	714–724	NEMVIGWKPPK	38	26
28	731–757	ILGYFLDQHDSEELDWHAVNQQPIPTR	12	
29	761–773	VSDLHEGHFYEFR	29	
30	796–809	EWTMPQPGPPYDVR	43	26
31	863–870	VSDLQPGK	42	15
32	935–944	DYKGPLDPQR	55	23
33	956–993	VILKEPGLEDLGTYSVIVTDADEDISASHTLTEEELEK	25	
34	960–993	EPGLEDLGTYSVIVTDADEDISASHTLTEEELEK	17	
35	1008–1019	LISGWNIDILER	64	45
36	1024–1030	LWLEVEK	14	20
37	1031–1044	LSPAAELHLIFNNK	52	55
38	1045–1052	EIFSSPNR	25	20
39	1045–1053	EIFSSPNRK	26	28
40	1053–1058	KINFDR	26	22
41	1061–1075	GLVEVIIQNLSEEDK	52	
42	1061–1086	GLVEVIIQNLSEEDKGSYTAQLQDGK	33	21
43	1089–1102	NQITLTLVDDDFDK	72	96
44	1089–1105	NQITLTLVDDDFDKLLR	60	23
45	1118–1129	QGPYFERPLQWK	16	14
46	1151–1157	FQWFFQR	22	23
47	1189–1218	AMVSDDRGEDDTILDLTGDALDAIFTELGR	88	32
48	1219–1228	IGALSATPLK	62	57
49	1229–1237	IQGTEEGIR	46	56
50	1244–1251	YYNVEYMK	40	30
51	1252–1257	TTWFHK	16	
52	1269–1284	TGTTLDEIWLHILDPK	19	21
53	1291–1299	YTLEIAAGK	37	35
54	1303–1322	QLSTDLSGQAFEDAMAEHQR	40	38
55	1325–1331	TLAIIEK	17	24

Ion scores (Mascot MS/MS ion scores) are shown for each peptide identified in each band. The entire length of MYOM3 is 1437 aa. The obtained peptide coverage of the MYOM3 suggests that both fragments have similar C-terminal end but different N-terminus. Minimum molecular weights of the fragments based on the positions of the most N-terminal and C-terminal identified peptides are equal to 121 kDa for the upper fragment (1077 aa) and 96 kDa (855 aa) for the lower. Thus estimated MW of the fragments fit well with the positions of the fragments on the SDS-PAGE (130 and 100 kDa respectively).

**Figure 2.**
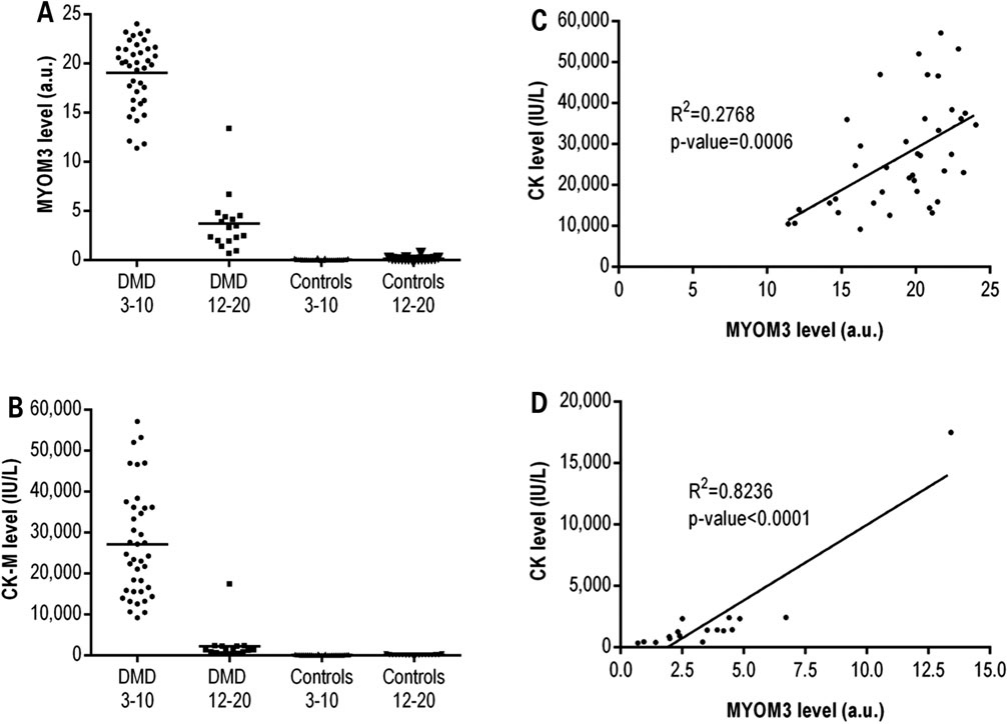
Expression levels of serum MYOM3 fragments (**A**) and CK (**B**) in sera from the entire US cohort including 39 young and 17 older DMD patients as well as 29 young and 18 older healthy controls. To measure levels of the MYOM3 fragments, 50 µg of serum proteins were analysed by Western blot, then band intensities were quantified and expressed in arbitrary units (a.u). The CK enzyme activity in serum is expressed in international units per litre (IU/L). (**C**) Linear regression analysis between serum levels of the MYOM3 fragments and CK for young patients. (**D**) Linear regression analysis between serum levels of the MYOM3 fragments and CK for older DMD patients. The linearity of the response by Western blot for MYOM3 is demonstrated in Supplementary material, Figure S2.

We next compared the levels of the MYOM3 fragments and CK in all 103 subjects from the US cohort. The serum expression levels of both MYOM3 fragments were determined by Western blot analysis and CK assessed by measuring its enzymatic activity (Fig. 2). In accordance with the mass spectrometry data, results showed that expression levels of both, CK and the MYOM3 fragments, were much higher in young DMD patients compared to the respective healthy controls (ratio DMD/Control: MYOM3 = 284 and CK = 193) (Fig. 2A and B). In older DMD patients, the expression levels of CK and MYOM3 fragments were respectively 14 and 5 times lower than in young DMD patients. Of note, in two outlier patients of 16 and 20 years old the levels of the MYOM3 fragments and CK were lower than the maximal values in the respective healthy controls. The decrease of these proteins with patient's age can be explained by the severe loss of total muscle mass due to the advanced stage of the disease.

Importantly, even if both proteins were able to discriminate DMD patients and healthy controls, there were less inter-individual variations in MYOM3 fragment levels compared to CK levels. While the CK levels in the young patients varied from 9000 to 60 000 IU/L (mean 27 130 IU/L ± 13 130), the values for MYOM3 fragments remained between 11 a.u. and 24 a.u. (mean 19 a.u. ± 3). The low correlation observed between the levels of serum CK and MYOM3 fragments in the group of young patients (*R*^2^ = 0.28) indicates that different physiological mechanisms may account for the secretion/stability of these proteins at this age (Fig. 2C). Conversely, these two biomarkers were well correlated in older patients (Fig. 2D).

### MYOM3 fragments are specifically present in sera from animal models of DMD

**Figure 3.**
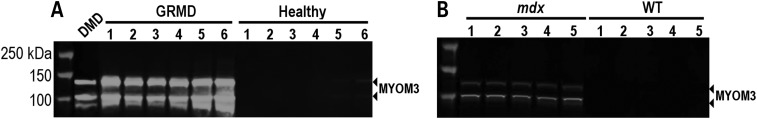
MYOM3 fragments are specifically present in sera from animal models of DMD. (**A**) Western Blot analysis of serum from GRMD and healthy dogs. GRMD # 1–4: two months old; # 5–6: 18 months old. Healthy # 1–4: two months old; # 5–6: 18 months old dogs. DMD: control serum from DMD patient. (**B**) Western Blot analysis of serum from 6 months old *mdx* and WT mice. WT: C57/BL10 strain.

The levels of MYOM3 fragments were quantified in two animal models of DMD: golden Retriever muscular dystrophy (GRMD) which has a severe phenotype similar to DMD patients (30) and dystrophin-deficient *mdx* mice. Western blot analysis of GRMD and *mdx* sera revealed the presence of two bands migrating at the same positions as human MYOM3 fragments (Fig. 3A and B). Importantly, the abundance of these fragments was 100 times higher than in the healthy control dogs. Whereas the level of the MYOM3 fragments in human DMD samples decreased with age, expression of these fragments was very similar in the serum of 2 and 18 months old ambulant GRMD. We hypothesize that the high MYOM3 levels in elder GRMD can be due to the ambulant state of the dogs. Age-independent expression of the MYOM3 fragments could be an advantage for utilization of this biomarker in gene therapy studies conducted in dogs.

### MYOM3 fragments are elevated in sera of LGMD2D patients and mouse models of LGMDs

**Figure 4.**
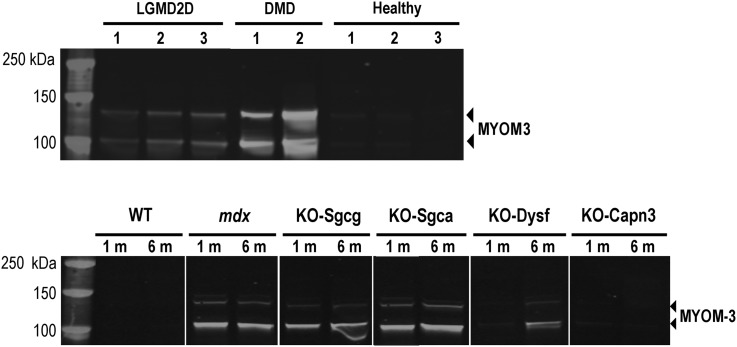
Upper panel: Western blot analysis of the MYOM3 fragments in serum from 3 LGMD2D patients (#1 is 35, #2 is 23 and #3 is 24 years old). Serum from two DMD patients (group G1) and three healthy individuals (group G4) were used as controls. Lower panel: Western blot analysis of the MYOM3 fragments in serum from mouse models of different muscular dystrophies at 1 and 6 months of age. WT: C57BL/6J mouse; *mdx* (model for DMD); KO-Sgcg: model for LGMD2C; KO-Sgca: model for LGMD2D; KO-Dysf: model for LGMD2B; KO-Capn3: model for LGMD2A.

The presence of the MYOM3 fragments was also analysed in serum samples of three patients with α-sarcoglycanopathy (LGMD2D). Fragments of the same length (100 and 130 kDa) were detected at elevated levels in all these patients. Overall, the level of these fragments in LGMD2D patients was lower compared to their intensity in young DMD patients (Fig. 4, upper panel).

The following mouse models of limb-girdle muscular dystrophies were included in this study: KO-Calpain 3 (models for LGMD2A) (31), KO-Dysferlin (models for LGMD2B) (32), KO-Sgcg (models for LGMD2C) (33) and KO-Sgca (models for LGMD2D) (34). These mouse models are congenic strains on the genetic background of the C57BL/6J mouse, which was included in the study as their wild-type (WT) control. Taking into consideration the muscle impairment and time of disease onset, these mouse models can be classified in terms of decreasing order of severity: KO-Sgca, KO-Sgcg, KO-dysf and KO-Capn3. Serum from these mouse models was collected at 1 and 6 months of age, corresponding to the early and advanced stages of the dystrophies, and the levels of the MYOM3 fragments were compared by Western blot. The highest levels of serum MYOM3 fragments were observed in the three mouse models with perturbations in the DAPC (Fig. 4, lower panel). In KO-Dysf mice, these fragments were barely detectable at 1 month of age and then increased at 6 months, reflecting the aggravation of the disease at this age. MYOM3 fragments were hardly detectable in KO-Capn3 mice at any age.

### In *mdx* mice, the MYOM3 fragments are expressed early, demonstrate less inter-individual variability and are less sensitive to physical exercise compared to CK

**Figure 5.**
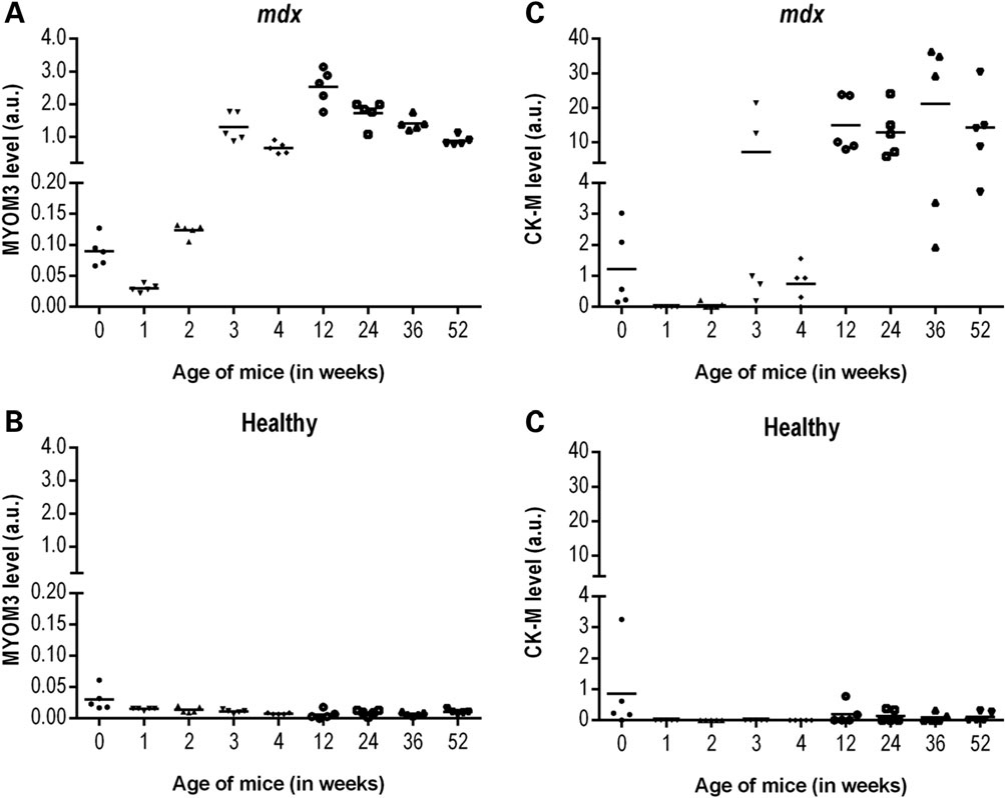
Levels of the MYOM3 fragments (**A**, **B**) and CK-M (**C**, **D**) in serum from healthy (B, D) and *mdx* (A, C) mice at different ages as estimated by Western blot analysis. Intensity of the bands (in arbitrary units, a.u.) on different gels was normalized by the respective bands of the positive control (50 µg of serum proteins from the same *mdx* mouse present on each gel). Fifty micrograms of serum proteins were used for the analysis. Age 0 corresponds to newborn mice. Estimation of the CK-M level by Western blot analysis correlated well with the CK activity (Supplementary material, Fig. S3).

In order to identify the earliest time point when the serum MYOM3 fragments are detectable, we investigated sera from *mdx* mice of different ages (from birth to 1-year-old). The MYOM3 fragments were detected in *mdx* mice at birth, with a small decrease in their levels at 1 week of age and followed by a rise in abundance at 3 weeks (Fig. 5A). Importantly, the levels of these fragments in the age-matched control mice was lower at all ages tested (Fig. 5B). The kinetics of the MYOM3 fragment abundance in the serum of *mdx* mice correlates with the timing of an acute phase of muscle necrosis generally occurring at 3–4 weeks of age, followed by an apparent stabilization of the muscle phenotype (35). The kinetics of serum CK levels in *mdx* mice were different from that of the MYOM3 fragments during the first weeks of age. Consistent with previous studies (36,37) serum CK was elevated in newborn mice, but then became undetectable during the 1st and 2nd week of age (except for 1 mouse), rising again at 3 and 12 weeks followed by a stabilization (Fig. 5C). In healthy mice, serum CK was also slightly elevated in newborns and 12- and 24-week-old animals (Fig. 5D). Importantly, less variation was observed in the levels of MYOM3 fragments in mice of the same age compared to the CK (maximum fold change two for MYOM3 versus 110 for CK). The difference in age-dependent expression patterns between serum CK and MYOM3 fragments in *mdx* mice is probably related to different mechanisms of bioprocessing of these proteins, especially during the early phases of disease.

**Figure 6.**
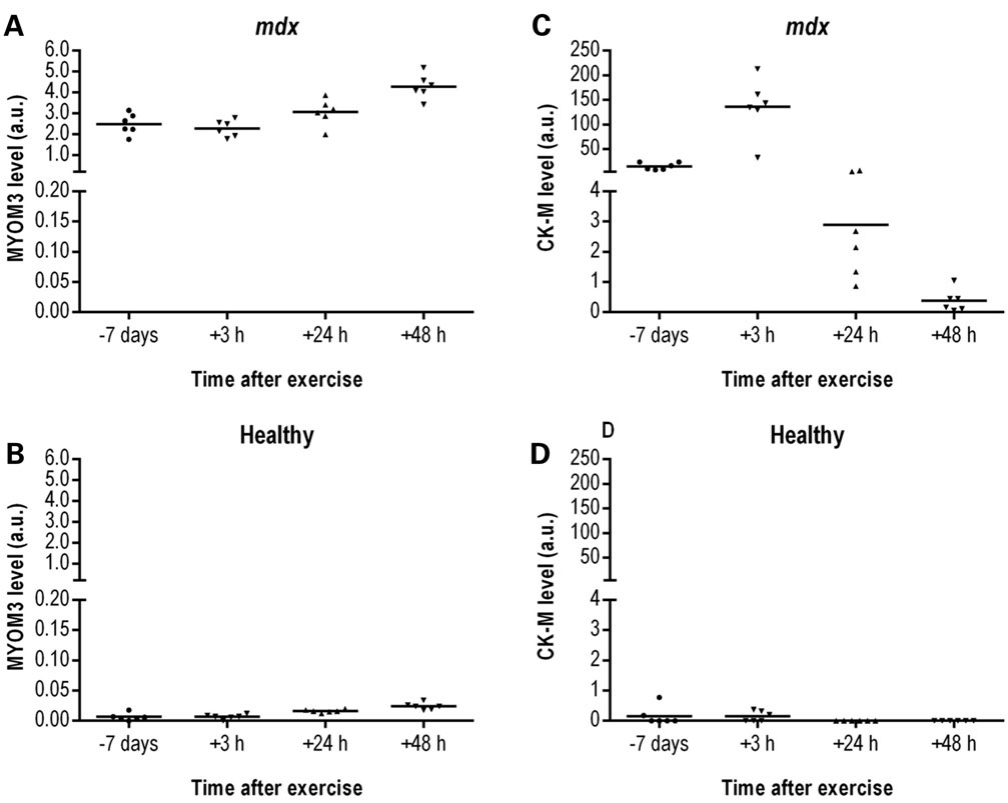
Levels of the MYOM3 fragments (**A**, **B**) and CK-M (**C**, **D**) in serum from healthy (B, D) and *mdx* (A, C) mice at different time after physical exercise estimated by Western blot analysis. Band intensity on different gels was normalized by the respective bands of the positive control (50 µg of serum proteins from the same mdx mouse present on each gel). Fifty micrograms of mouse serum were used for the analysis.

To assess the impact of physical exercise on the serum levels of the MYOM3 fragments and CK, WT and *mdx* mice were subjected to downhill running for 30 min. This exercise regimen is often used to increase muscle injury and worsen the *mdx* phenotype (38,39). Sera were collected 7 days before and 3, 24 and 48 h after exercise. Importantly, while in *mdx* mice CK concentration peaked at 3 h post-exercise (up to 10-fold increase) followed by a substantial decrease (Fig. 6C), physical exercise had relatively little impact on the serum levels of the MYOM3 fragments (less than 2-fold increase 48 h post-exercise) (Fig. 6A). Interestingly, in healthy mice, there was a slight increase in the levels of the MYOM3 fragments 24 and 48 h after exercise, even though the maximum level of the fragments in healthy mice was 50-fold less than in *mdx* mice (Fig. 6B). Serum CK levels were variable in healthy mice without noticeable correlation with physical exercise (Fig. 6D). Given that MYOM3 is predominantly expressed in slow and intermediate speed (type I and IIa) skeletal fibres (29) which are less affected in DMD relative to fast myofibres (40), it is possible that the difference in the kinetics of these biomarkers is partially due to the differential sensitivity of these muscle fibre types to exercise-induced damage.

### MYOM3 fragments enable monitoring of pharmaco- and gene-therapy treatment efficacy

The presence of the MYOM3 fragments in serum of DMD and LGMD2D patients and their respective mouse models prompted us to evaluate the utility of these biomarkers for monitoring the response to experimental therapies in *mdx* and KO-Sgca mice.

**Figure 7.**
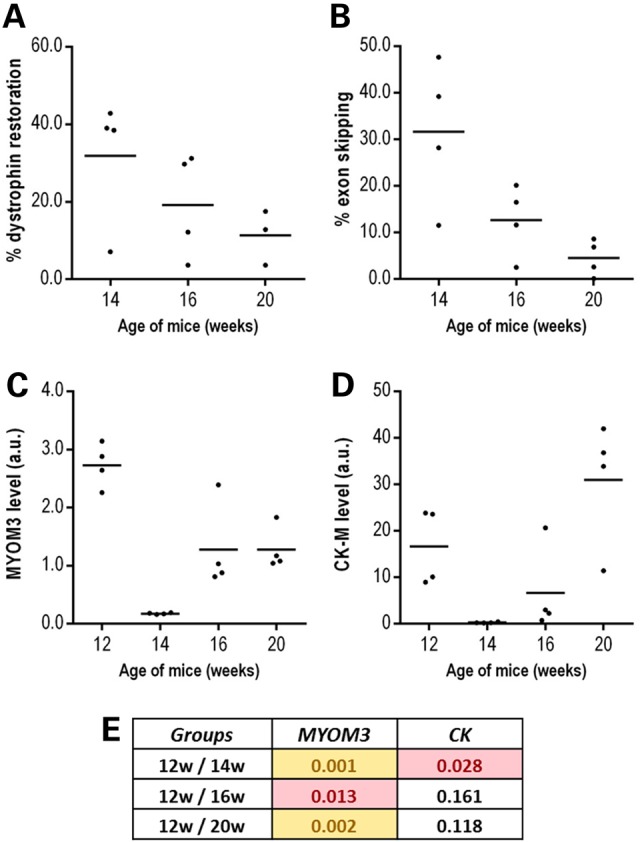
Effect of antisense oligonucleotide-mediated exon-skipping therapy in mdx mice on the serum levels of the MYOM3 fragments and CK-M. Twelve 12-week-old male *mdx* mice were treated with a single 12.5 mg/kg intravenous dose of Pip6a-PMO. Two, 4 and 8 weeks after injections the animals were sacrificed by groups of four mice and quadriceps and blood samples were taken for analysis. Dystrophin restoration in quadriceps muscles was monitored by Western blot analysis (**A**) and the efficiency of exon skipping by qPCR analysis (**B**) (See Materials and Methods section for the details). Levels of the MYOM3 fragments (**C**) and CK-M (**D**) were measured by Western blot analysis, and then band intensities were quantified and expressed in arbitrary units (a.u). Additional data confirming the robustness of dystrophin quantification are presented in Supplementary material, Figure S4. (**E**): Raw *P*-values (Student's *t*-test) for the comparison of MYOM3 and CK levels between different groups of mice at different time points. The values below the threshold 0.01 are in yellow, and below 0.05 are in pink.

Restoration of dystrophin expression in *mdx* mouse muscles was achieved by a single administration of an arginine-rich cell-penetrating peptide (CPP) conjugated to a phosphorodiamidate morpholino oligonucleotide (PMO) that efficiently induces skipping of exon 23 and restores dystrophin protein expression and muscle function (41,42). In order to evaluate the impact of the restoration of dystrophin expression on the serum levels of MYOM3 fragments and CK, quadriceps femoris muscles and blood samples from treated *mdx* were collected 2, 4 and 8 weeks post-injection. Quadriceps and blood samples from non-treated *mdx* and WT control 12-week-old mice were taken as controls. In accordance with the previously published data (41–43), the restoration of the dystrophin expression and percentage of exon skipping in quadriceps were between 10 and 45% 2 weeks after injections, followed by a decrease at later time points (Fig. 7A and B). In a good agreement with the restoration of dystrophin levels, two weeks after injection the levels of the MYOM3 fragments in treated *mdx* mice substantially decreased (without reaching the level in the control mice) and then gradually increased over time (Fig. 7C). In contrast to the MYOM3 fragments, CK levels did not reflect restoration of dystrophin expression. Thus, 2 weeks after injection the level of serum CK was lower in treated *mdx* mice compared to WT control mice (Fig. 7D), while dystrophin expression did not exceed 50% at that time. Moreover, 8 weeks after the treatment, when the estimated level of dystrophin-positive fibres was around 10%, CK levels were higher in treated than in non-treated *mdx* mice. Different behaviour of the MYOM3 fragments and CK after partial restoration of dystrophin expression may reflect the capacity of these biomarkers to differentially reveal intracellular process such as microparticle turnover (44) or increased myofibrillar protein catabolism (45,46).

To restore α-sarcoglycan expression in KO-Sgca mice, we used recombinant adeno-associated virus rAAV2/8 vector. Control C57BL/6J mice received an intravenous injection of PBS and four groups of KO-Sgca mice received intravenous injections of either PBS or low (1e11 vg), medium (5e11 vg) or high (1e12 vg) doses of rAAV2/8 coding for hSGCA. Mice were monitored for 3 months after the treatment. The following assays were compared in order to define the most appropriate for the follow-up of the treatment: histological analysis of muscle biopsies (HPS staining and restoration of the sarcoglycan complex); total physical force 3 months after the treatment (1 week before animal sacrifice); biweekly analysis of serum CK and MYOM3 fragments levels.

**Figure 8.**
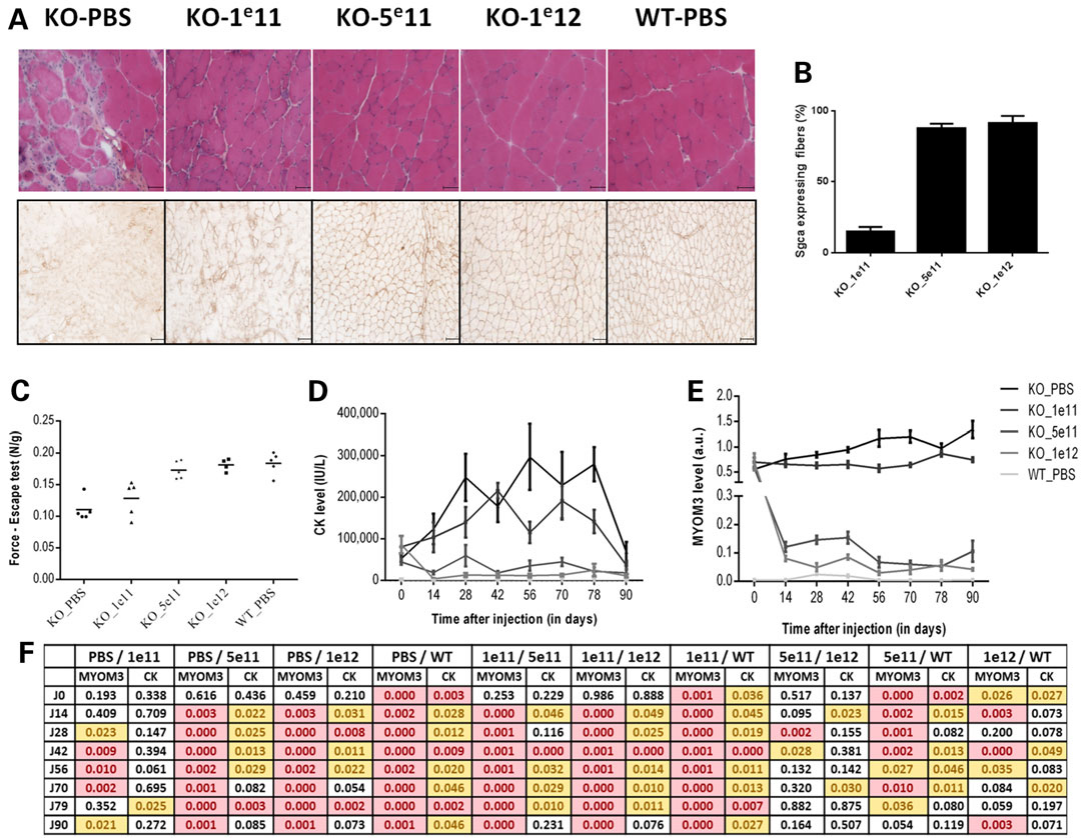
Comparison of different assays for the follow-up of the gene therapy treatment in KO-Sgca mice. (**A**) Histological analyses (upper panel: HPS stain; lower panel: immunodetection of α-sarcoglycan) of gastrocnemius muscles after treatment with increasing doses of rAAV coding for huSgca (1e11, 5e11 and 1e12 vg). (**B**) Quantitative analysis of α-sarcoglycan positive fibres after the treatments. (**C**) Restoration of muscular strength (escape test) 83 days after the treatment. (**D**) Serum CK and (**E**) MYOM3 fragments levels at different time after the treatment (mean ± SEM). (**F**) Raw *P*-values (Student's *t*-test) for the comparison of MYOM3 and CK levels between different groups of mice at different time points. The values below the threshold 0.01 are in pink, and below 0.05 are in yellow. PBS, 1e11, 5e11 and 1e12: KO-Sgca mice injected with PBS or the respective dose of the vector. WT: C57BL/6J control mice injected with PBS. Levels of the MYOM3 fragments and CK-M were estimated biweekly by Western blot analysis.

Histological analysis of the gastrocnemius muscles demonstrated restoration of the complex in 5–30% (mean 15.6 ± 8.4), 60–100% (mean 79.2 ± 16.7) and 84–100% (mean 94.6 ± 8.8) of fibres after low, medium and high rAAV dose treatments, respectively (Fig. 8A and B). Importantly, by assessing the expression level of α-sarcoglycan (determined by immunostaining) the KO-Sgca, low, medium and WT mice could be clearly distinguished. However, no statistically significant difference was found between medium and high rAAV doses by this method. Importantly, this analysis is highly laborious, and the size of the biopsies makes it unsuitable for the follow-up of the therapeutic effect in small animals.

Similar to histological analysis, the conventional whole body tension (WBT) method is an end-point assay because mice become accustomed to the protocol (47). The WBT method was only able to discriminate two clusters of animals: (1) KO-Sgca mice injected with PBS or low dose of rAAV and (2) control C57BL/6J mice and KO-Sgca mice injected with medium or high doses of rAAV (Fig. 8C).

A threshold 3000 IU/L of CK clearly separates KO-Sgca mice injected with PBS from all other experimental groups (Fig. 8D). Nevertheless, when applying the Student's test (*P*-value threshold < 0.01), differences only between few time points/injection doses appeared as statistically significant (Fig. 8F). Changing of the *P*-value threshold to <0.05 permits to distinguish more experimental groups of mice (Fig. 8F). Lower CK levels in all groups of mice at day 90 (one week after the total force measurements) (Fig. 8D) could be explained by the fact that an increase of CK levels after physical exertion is followed by a substantial decrease persisting for 2 weeks (48).

Inter-individual variations of the MYOM3 fragment levels were lower compared to serum CK in the case of all experimental groups (Fig. 8D and E). In accordance with a previous study showing progressive development of muscular dystrophy in KO-Sgca mice (34) the levels of the MYOM3 fragments in the control mice injected with PBS increased gradually with age (Fig. 8E). Even the lowest dose of rAAV (1e11 vg) stabilized the MYOM3 fragment levels, while medium and high doses reduced MYOM3 fragment levels 5-fold and 8-fold, respectively. Due to the low inter-individual variability, measurement of the MYOM3 fragments enabled nearly all groups of mice to be distinguished with either of the thresholds (*P* < 0.01 or 0.05) at the majority of time points (Fig. 8E and F). Furthermore, MYOM3 fragment abundance was better correlated (*R*^2^ = 0.71) with muscle force as measured by the escape test compared with CK (*R*^2^ = 0.59) (Supplementary material, Fig. S1).

## Discussion

In this study, the protein composition of serum samples from DMD patients and their age-matched healthy controls were compared using a label-free proteome profiling strategy. Using this approach, 37 proteins were identified as differentially expressed between young DMD patients and their age-matched controls. Here we discuss the utility of these proteins as potential diagnostic or therapeutic biomarkers for muscular dystrophies.

### Presence of CK and other cytosolic proteins in the serum of DMD patients

Despite a large number of proteins with altered levels reported in the serum of muscular dystrophy patients (49), CK is currently the most widely used diagnostic tool in the early stages of DMD and LGMD diagnosis (25,50). CK is predominantly a cytosolic protein: only a small amount of cytosolic CK (5–10%, depending on muscle fibre type) is specifically bound to the myofibrillar M-band, whereas the soluble major fraction can be extracted by buffers of physiological ionic strength (51–53). The widespread utilization of this biomarker can be explained by the low cost of its assessment, the high sensitivity of existing CK assays, the excellent negative predictive value and also by a textbook explanation for the elevation of this protein in the blood of DMD patients: leakage due to the increased fragility of the sarcolemma in myofibres in the absence of dystrophin. While the membrane of normal muscle fibres can be quickly repaired (54), perturbation of the DAPC could increase repair time thus leading to the leakage of cytosolic proteins into the bloodstream. Increased levels of glycolytic enzymes (pyruvate kinase, L-lactate dehydrogenase, β-enolase, fructose-bisphosphate aldolase A, glycogen phosphorylase, glucose-6-phosphate isomerase, triosephosphate isomerase) which are also considered to be soluble constituents of the cell (55,56) in the bloodstream of patients support this leakage hypothesis. It is highly probable that most of the cytosolic proteins with increased levels in DMD patient serum could be used as biomarkers in DMD equally to CK. Thus, excellent correlation between the levels of CK and pyruvate kinase was found in sera from DMD and Becker patients (23). Nevertheless, we cannot exclude dysregulation of vesicle trafficking due to lack of dystrophin as a possible source of cytosolic/cytoskeletal proteins in DMD patient serum (44).

### Secreted proteins

Of the 24 proteins enriched in DMD patient sera only haptoglobin, which is known to be elevated during inflammatory processes (57), is known to be secreted. Intriguingly, all 13 proteins with decreased levels in DMD patient sera were either secreted or membrane components. The secreted proteins exhibited lower fold changes between DMD and healthy controls compared to cytosolic or structural proteins (maximum 5-fold versus 250-fold, respectively) and higher *P*-values. However, these proteins could be useful in DMD prognosis if they appear to be correlated with clinical outcome, natural history or therapeutic response, and further studies are needed to define their roles in disease development. Interpreting the biological meaning of these secreted factors by using the Ingenuity software (http://www.ingenuity.com) indicated the involvement of lipid and carbohydrate metabolism in the disease. In accordance with these findings, recent publications described perturbation in lipid (58) and glycogen metabolism (59) in human DMD myoblasts and *mdx* mice respectively.

### Cytoskeletal proteins

Of the 37 proteins with altered levels at the early stage of the disease, seven were cytoskeletal (MYOM2, MYOM3, Myosin-7, Filamin-C, Titin, Vinculin and Tropomyosin β chain). All these proteins were upregulated in sera from DMD patients. Importantly, increased levels of fragments of MYOM3, Titin and Filamin-C were recently demonstrated in sera of a small cohort of DMD patients (49). The presence of the structural myofibrillar proteins in the blood may be due to the accelerated remodelling of muscle fibres in DMD patients accompanied by an increased secretion of protein fragments, or by the catabolic process of the damaged fibres and muscle necrosis. Independently of the mechanisms underlying the appearance of these proteins in the bloodstream, we sought to evaluate whether the levels of myofibrillar proteins in serum could be used for treatment monitoring in DMD, LGMD2D and potentially in other forms of muscular dystrophy. Based on the high fold change and low *P*-value between DMD and healthy controls, and also on the availability of antibodies, MYOM3 protein was chosen for this further analysis.

### MYOM3 fragments as biomarkers for therapeutic outcome measurements

Myomesins are the principal components of the cytoskeletal structure called the M-band that cross-links filamin-C and titin filaments in the middle of the sarcomere (60). There are three closely related structural forms of this protein: myomesin (MYOM1) and M-protein (MYOM2) and myomesin 3 (MYOM3). According to amino acid alignment, the identity level between MYOM3 and MYOM1 or MYOM2 is about 40%, and all three proteins share the same domain arrangement (29). It was suggested, that similarly to titin, myomesin is a molecular spring whose elasticity guards the stability of the sarcomere (61,62). Expression of MYOM3 was found mainly in intermediate speed fibres (type IIa) of skeletal muscle, while fast fibres express more MYOM2 and MYOM1 is expressed in all muscle fibres (29).

Presence of the two fragments of 100 and 130 kDa in the sera of DMD patients as well as LGMD2D patients and in different animal models of DMD (*mdx* mouse, GRMD dog) and other forms of muscular dystrophy indicates a conserved mechanism of MYOM3 proteolysis and release across species and diseases. This mechanism is possibly linked to the activation of calcium-dependent proteolysis pathways such as those involving the ubiquitous calpains (63). Importantly, there was a clear difference in the time-course of cytosolic CK and structural MYOM3 proteins appearance in the bloodstream of *mdx* mice after physical exercises. In accordance with published data, the CK levels varied as much as 20-fold with a peak at 3 h after exercise (48), while the amounts of the MYOM3 fragments increased only by 2-fold, peaking at 2 days after exercise. Moreover, serum CK was present as a complete protein, while MYOM3 was present as two fragments. To explain the different behaviour of these cytosolic and structural proteins, we suggest that the minor injuries that are reparable by the membrane reparation machinery (64) are sufficient to allow the escape of cytosolic proteins into the bloodstream. More severe damage can lead to the failure of membrane repair and trigger myofibre necrosis (65) followed by proteolysis and solubilization of structural myofibrillar proteins. Thus, the progressive rounds of myofibre necrosis followed by rounds of muscle fibre regeneration characteristic of DMD muscles can lead to the appearance of both, cytosolic proteins and structural myofibrillar protein fragments in the bloodstream, while low level membrane damage results in the release of cytosolic proteins only. Low correlation between the levels of the MYOM3 fragments and CK in young patients favours this hypothesis. Nevertheless, we cannot exclude that the abnormal presence of cytoskeletal proteins in sera of DMD patients is due to dysregulation of vesicle trafficking in the absence of dystrophin (44). The differences in the biogenesis of these biomarkers suggests that structural proteins can be a complementary to the cytosolic proteins such as CK with the former reflecting more severe muscle damage while being less sensitive to occasional myofibre leakage. However, the behaviour of serum biomarkers cannot be simply predicted on the basis of cytosolic or structural classification. Thus, products of proteolysis of another structural protein titin can be found in serum (our data not shown) and urine of *mdx* mice as early as 3 h after physical exercise (22). The different behaviour of titin and MYOM3 can be due to the particular role of titin as a sensor of mechanical load (66).

Comparison of MYOM3 fragments with three other assays (biopsy, restoration of physical force and CK) in models of two different muscular dystrophies, *mdx* (dystrophin deficient) and KO-Sgca (α-sarcoglycan deficient), demonstrated the superiority of MYOM3 fragments for the follow-up of gene therapy treatments relative to other assays. The advantages of the MYOM3 fragments compared to CK are their lower inter-individual variability between the patients of the same age, better correlation with the reconstitution of the dystrophin-associated protein complex and muscle force restoration. The critical advantages of the MYOM3 fragments compared to the histological analysis of biopsies are that they are less invasive and provide information concerning body-wide muscle integrity. MYOM3 was the most efficient biomarker for distinguishing the five groups of KO-Sgca mice treated with different doses of rAAV vector. Importantly, for none of the recently proposed biomarkers based on quantification of serum miRNA (19,20,67) or proteins (28,68), a therapy-dose-dependent return to normal values has been shown so far. This is a crucial advantage of MYOM3 compared to other proposed proteins or miRNAs for which this remains to be shown. Even if miRNAs could potentially provide a similar degree of qualitative or quantitative information concerning therapy response, this method remains more laborious and costly. For the moment however, primary data are still missing.

Taken together, our data demonstrate that MYOM3 fragments are promising candidate biomarkers for monitoring therapeutic outcomes in DMD and other muscular dystrophy patients.

## Materials and Methods

### Human sample collection

The human studies were conducted according to the principles of the declaration of Helsinki “Ethical Principles for Medical Research Involving Human Subjects”. Serum samples from a cohort of 39 young (3–10 years old) and 17 older (12–20 years old) DMD patients as well as 29 young (3–10 years old) and 18 older (12–20 years old) healthy individuals were collected at the Cincinnati Children's Hospital Medical Center USA (US cohort) as part of ADNA (Avancées Diagnostiques pour de Nouvelles Approches thérapeutiques) project (http://www.institut-merieux.com/projetssante_adna.php). All young patients were ambulant, while all older patients were wheelchair bound. None of the patients was treated with corticosteroids, and all patients with a CRP level higher than 1 mg/dl were excluded from the study. Age, genetic alterations, CK and MYOM3 levels of DMD patients in the cohort are shown in Supplementary Material, Table S1. The study protocol and Informed Consent was approved by the Institutional Review Board (IRB) at Cincinnati Children's Hospital Medical Center. Informed Consent was obtained from all subjects prior to the study. The conduct of the study conforms to all applicable human subjects research regulations. We could provide documentation upon request. Serum samples from three LGMD2D patients were collected at the Neuromuscular Research Center (University Hospital of Tampere, Finland) during standard day-care consultation. All three patients were homozygous for 299CGC > TGC (R77C) mutation in the alpha-sarcoglycan gene. Patients of 35 and 24 years old were non-ambulant, while 23 years old patient was ambulant at the time of the blood draw. After collection, samples were centrifuged twice immediately (10 000 × *g*, 10 min) and serum was stored at −80°C.

### Protein quantification and measurements of CK activity

Protein concentration was determined using the Bio-Rad Protein Assay Dye Reagent (Bio-Rad) according to the manufacturer's instructions with bovine serum albumin as a standard. Measurements of total CK activity were performed using the Vitros DT60 II Chemistry System according to the manufacturer's instructions (Ortho-Clinical Diagnostics).

### Serum depletion

Depletion of the 12 most abundant serum proteins (alpha 1-acid glycoprotein, alpha 1-antitrypsin, alpha 2-macroglobulin, albumin, apolipoprotein A-I, apolipoprotein A-II, fibrinogen, haptoglobin, IgA, IgG, IgM and transferrin) was performed with the Proteome purify 12 Human Serum Protein Immunodepletion kit (R&D Systems) according to the manufacturer's instructions with some modifications. Briefly, 1 ml of immunodepletion resin was mixed with 10 µl of pooled serum diluted with PBS to a final volume of 500 µl and incubated for 1 h at room temperature (RT). Depleted serum was collected after centrifugation (1000 × *g*, 2 min) in Spin-X Filter Units and proteins were 5-fold concentrated using Amicon Ultra-2 Centrifugal Filter Units (cut-off 3 kDa; Millipore) following the manufacturer's instructions.

### MYOM3 fragments purification

MYOM3 fragments were purified from 500 µl of DMD patient serum by immunoprecipitation with anti-MYOM3 antibodies (Proteintech: 17692-1-AP) immobilized on A/G sepharose. After five washing with PBS supplemented with 0.5% of Triton X-100 immunoprecipitated proteins were separated by SDS-PAGE and Coomassie stained. Two bands migrating at the same positions as on the Western blot analysis were taken for the mass spectrometry analysis using LTQ Velos Orbitrap mass spectrometer (Thermo Fisher Scientific).

### Mass spectrometry

For mass spectrometry analysis, 10 µg of depleted serum proteins were solubilized in a total of 123 µl of the reaction mixture containing 4 M urea, 1.5 M thiourea and 50 mm tris–HCl pH 8.3. Proteins were reduced with 10 mm dithiothreitol for 30 min and then alkylated with 55 mm iodoacetamide for 20 min. Alkylated proteins were first digested with 500 ng of endopeptidase lys-C (Wako) for 3 h at RT. Then, the mixture was adjusted to 235 µl with MilliQ-water and treated with 500 ng of trypsin (Sequence Grade Trypsin, Promega) for 16 h at RT. Enzymatic activity was stopped by addition of formic acid to 3% final concentration and samples were stored at −20°C until use. The peptide mixture was desalted using ZipTip_µ-C18_ Pipette Tip (Millipore) and separated with an Easy nano-LC Proxeon system (Thermo Fisher Scientific) equipped with a reversed phase C18 column (Easy-Column Proxeon C18, L 10 cm, ID 75 μm). Eluates were monitored by a LTQ Velos Orbitrap mass spectrometer (Thermo Fisher Scientific) and tandem MS (MS/MS) data were processed with Proteome Discoverer 1.4 software (Thermo Fisher scientific) coupled to an in house Mascot search server (Matrix Science, 2.3.2 213 version) using SwissProt database as described previously (22). The relative abundance of each protein identified in serum from DMD or healthy patients was estimated by label-free quantification using the Progenesis LC-MS software (Nonlinear Dynamics, 4.0 version).

### Western blot

Protein samples were separated by SDS-PAGE electrophoresis (4–12% gradient, NuPAGE Novex Bis-Tris Gel 1.0 mm, Life Technologies) and transferred onto Protran Premium membrane (nitrocellulose, GE Healthcare). Fifty micrograms (1 µl of serum) of human, mouse or dog serum protein were loaded per lane. Antibodies against MYOM3 (1:1000, Proteintech: 17692-1-AP) and the CK-M (1:500, Santa Cruz: sc-15161) were used as primary antibodies followed by incubation with the corresponding IRDye-800CW-conjugated antibodies (1:10 000, LI-COR Biosciences) according to the manufacturer's instructions. Infrared fluorescence of the secondary antibodies was read on an Odyssey Imaging System (LI-COR Biosciences). Band intensities were measured by the Odyssey application software (LI-COR Biosciences, Image Studio Lite 4.0 Version). The following antibodies were not efficient in Western blot analysis of human serum: anti-Myomesin 3 sc-165061; anti-Myomesin 2: sc-30384; sc-30385; sc-50435; anti-Myosin: ABIN502241; ABIN616957; sc-53089; sc-20641; sc-32732; sc-53096.

### Animal experimentations

Animal experimentations were conducted in accordance with the European guidelines for the protection of vertebrate animals used for experimental purposes (Directive 2010/63/EU of 22 September 2010) and for the mice treated with the oligonucleotide Pip6a-PMO, in accordance to procedures authorized by the UK home office. Blood samples were collected from ambulant male dogs (provided by the CEDS at Mézilles and Oniris at Nantes, France) from the lateral saphenous vein and from mice by retro-orbital bleeding or from the jugular vein. The following mouse strains were used: C57BL/6 and C57/BL10 control strains as well as *mdx* [model for DMD: (69)], and four knockout (KO) strains named KO-Capn3 [model for LGMD2A: (70)], KO-Dysf [model for LGMD2B: (32)], KO-Sgcg [model for LGMD2C: (33)] and KO-Sgca [model for LGMD2D: (34)]. Blood samples were centrifuged twice (10 000 × *g*, 10 min, 4°C) and serum samples obtained were stored at −80°C until use.

### Physical exercise of mice

Mice were placed on a treadmill (Treadmill Exer 6M, Columbus Instruments) to run at a downward inclination of 15° at speeds of 8 m/min for 5 min, followed by 12 m/min for 25 min. Blood samples were collected by retro-orbital bleeding and sera were stored at −80°C.

### Ageing in mice

Serum samples from 1-day up to 52-week-old mice (five healthy controls or five *mdx* per age) were stored at −80°C. Newborn as well as 12-, 24-, 36- and 52-week-old mice were euthanized after collection. The samples for the 1-, 2-, 3- and 4-week time points were collected from the same group of mice.

### Antisense oligonucleotide-mediated exon-skipping therapy of *mdx* mice

Twelve-week-old *mdx* mice were treated with a single 12.5 mg/kg tail vein injection of an arginine-rich CPP conjugated to a PMO, Pip6a-PMO (peptide RXRRBRRXRYQFLIRXRBRXRB coupled through an amide linkage at the 3′ of the oligonucleotide 5′-GGCCAAACCTCGGCTTACCTGAAAT-3′), synthesized and prepared in a sterile saline solution as described previously (42,67). Two, 4 and 8 weeks post-injection animals were sacrificed by groups of four mice and quadriceps and blood samples were taken for analysis. The blood at 12 weeks was collected by retro-orbital bleeding and at 14, 16 and 20 weeks through jugular vein (end-point bleeds) and the serum levels of the MYOM3 fragments and CK-M were monitored by Western blot analysis. Dystrophin restoration in quadriceps muscles was followed up by Western blot analysis and the efficiency of exon skipping by qPCR analysis.

To assess dystrophin protein restoration, quadriceps cryo-sections were lysed in buffer (75 mmol/l Tris–HCl (pH 6.5), 10% sodium dodecyl sulphate, 5% 2-mercaptoethanol and protease inhibitors) prior to centrifuging at 13 000 rpm for 10 min. After heating at 100°C for 3 min supernatant was fractionated on a 3–8% Tris-Acetate gel. Proteins were transferred and probed with a monoclonal anti-dystrophin (1:200, NCL-DYS1, Novocastra) and anti-vinculin (loading control, 1:100 000, hVIN-1, Sigma) antibodies. Secondary antibody IRDye-800CW goat anti-mouse was used at a dilution of 1:20 000 (LiCOR). Fluorescence was detected and quantified using the Odyssey imaging system. There was no saturation of signal using this methodology, thereby allowing for dystrophin protein quantification over a wide linear dynamic range. Dystrophin expression was quantitated using the dystrophin to vinculin ratio versus C57BL10 wild-type dystrophin expression level standards on each gel.

To investigate the duration of exon skipping after Pip6a-PMO administration, RNA was extracted from quadriceps cryo-sections using Trizol (Invitrogen). A total of 1 µg of RNA was reverse transcribed using the High Capacity cDNA RT Kit (Applied Biosystems, Warrington, UK) according to manufacturer's instructions. qPCR analysis was performed using 25 ng cDNA template and Taqman Gene Expression Master Mix (Applied Biosystems, Warrington, UK) on a StepOne Plus Thermocycler (Applied Biosystems, Warrington, UK). Levels of dystrophin exon 23 skipping was determined by multiplex qPCR of FAM-labelled primers spanning Exon 20-21 (Assay Mm.PT.47.9564450, Integrated DNA Technologies, Leuven, Belgium) and HEX-labelled primers spanning Exon 23-24 (Mm.PT.47.7668824, Integrated DNA Technologies, Leuven, Belgium). The percentage of dystrophin exon 23 transcript skipping was determined by normalizing exon 23-24 amplification levels to exon 20-21 levels.

### Gene therapy treatment of KO-Sgca mice

Recombinant adeno-associated virus 8 (rAAV2/8) vector was used to restore α-sarcoglycan expression in KO-Sgca mice. The production of rAAV was performed by dual infection of Sf9 cells with baculoviruses harbouring cDNA for Sgca under the desmin promoter and regulated by miR-142-3p (71) and AAV *rep2/cap8* genes (rAAV2/8). The purification was performed on immuno-affinity AVB sepharose medium (GE Healthcare) according to (72). Four groups of 5-week-old KO-Sgca mice (five mice per group, except four mice for the highest vector dose) were injected either with PBS or with increasing doses of rAAV [1e11, 5e11 and 1e12 viral genome (vg)]. Blood samples were collected biweekly for 3 months and levels of the MYOM3 fragments and CK were monitored by Western blot analysis and measurements of CK activity, respectively. Muscle force was measured by the escape test 1 week before sacrifice. Restoration of the sarcoglycan complex and muscle morphology was assessed by immunostaining and histological analyses.

### Evaluation of muscle force in mice

Mouse muscle force was evaluated by the WBT method or escape test (73) with some modifications. Mice attached to the tail with a thread connected to a tension transducer were placed on a platform facing the entrance of a 30 cm long tube. In response to pinching of the tail, mice try to escape within the tube thus raising a short peak of force (forward pulling tension, FPT) that is recorded. Five FPTs were recorded for each mouse. The body weight of each mouse was measured and the WBT was obtained by dividing the average of the five FPTs with the body weight.

### Statistical analysis

Statistical analyses were performed using the GraphPad Prism version 6.04. Data are expressed as mean ± SD if not otherwise specified. For comparisons between means, homogeneity of variances was assessed by Fisher–Snedecor's test and the Student's *t*-test (two-tailed) was applied. Pearson's correlation was used for correlation studies and data were analysed with a 95% confidence interval and *P* < 0.05 was considered significant.

### Histology and Sgca immunostaining

Cryosections (8 mm thickness) were prepared from frozen right and left gastrocnemius muscles. Transverse sections were processed for haematoxylin phloxine saffron (HPS) histological staining. Colorimetric immunodetection of Sgca was performed as previously described (74).

After digitization of immunostained biopsies (Axioscan, ZEISS) the total surface of the biopsies and the surface stained for α-sarcoglycan were quantified using the ImageJ sofware (version 1.47 g 64-bits, Rasband, W.S., ImageJ, U. S. National Institutes of Health, Bethesda, Maryland, USA, http://imagej.nih.gov/ij/, 1997–2014) and a customized script (available on demand). The percentage of Sgca positive fibres for a given biopsy was calculated using the following equation: (number of Sgca positive pixels on the biopsy area/surface in µm² of the biopsy area) divided by the same ratio obtained for healthy control (number of Sgca positive pixels on the biopsy area/surface in µm² of the biopsy of the control C57BL/6J mouse) and multiplied by 100.

## Supplementary Material

Supplementary Material is available at *HMG* online.

## Funding

This work was supported by the Association Française contre les Myopathies (AFM). This work is a contribution to ADNA (Advanced Diagnostics for New Therapeutic Approaches), a program dedicated to personalized medicine, coordinated by Institut Mérieux, supported and partially funded by the French public agency OSEO. Funding to pay the Open Access publication charges for this article was provided by Genethon.

## Supplementary Material

Supplementary Data
