# Supplementary material for: Serum proteomic profiling reveals fragments of MYOM3 as potential biomarkers for monitoring the outcome of therapeutic interventions in muscular dystrophies
Source: Hum Mol Genet. 2015 Jun 9;24(17):4916–32. doi: 10.1093/hmg/ddv214 (PMC4527491; doi:10.1093/hmg/ddv214)
Supplement: Supplementary Data [file supp_24_17_4916__index.html]

Serum proteomic profiling reveals fragments of MYOM3 as potential biomarkers for monitoring the outcome of therapeutic interventions in muscular dystrophies — Serum proteomic profiling reveals fragments of MYOM3 as potential biomarkers for monitoring the outcome of therapeutic interventions in muscular dystrophies — Supplementary Data 

# Serum proteomic profiling reveals fragments of MYOM3 as potential biomarkers for monitoring the outcome of therapeutic interventions in muscular dystrophies

## Supplementary Data

Supplementary Data

- Supplementary Data - Docx file
