# Supplementary material for: Serum proteomic profiling reveals fragments of MYOM3 as potential biomarkers for monitoring the outcome of therapeutic interventions in muscular dystrophies
Source: Hum Mol Genet. 2015 Jun 9;24(17):4916–32. doi: 10.1093/hmg/ddv214 (PMC4527491; doi:10.1093/hmg/ddv214)
Supplement: Supplementary Data [file supp_ddv214_ddv214supp_data.docx]

**Supplementary material**

**Supplementary Table 1.**

Age, genetic alterations, CK and MYOM3 levels of DMD patients in the cohort. None of the patients was treated with corticosteroids, and all patients with a CRP level higher than 1 mg/dl were excluded from the study. All young patients were ambulant, while all older patients were wheelchair bound.

| **Age** | ***Mutation*** | **CK-M** | **MYOM3** |
| --- | --- | --- | --- |
| 3.0 | deletion 46-50 | 57,167 | 21.7 |
| 3.2 | deletion 49-52 | 36,235 | 23.0 |
| 3.3 | deletion 49-50 | 46,671 | 21.5 |
| 3.4 | nonsense 11 | 29,567 | 16.3 |
| 3.5 | deletion 45-50 | 53,254 | 22.9 |
| 3.7 | deletion exon 45 | 23,084 | 23.2 |
| 3.9 | deletion 8-34 | 27,549 | 22.4 |
| 4.0 | deletion 45 | 22,444 | 19.8 |
| 4.1 | stop exon 23 | 47,012 | 17.6 |
| 4.2 | duplication 10-11 | 27,679 | 20.1 |
| 4.3 | deletion exon 44 | 52,095 | 20.2 |
| 4.3 | deletion 3-19 | 14,444 | 20.9 |
| 4.3 | deletion 48-50 | 21,780 | 19.6 |
| 4.5 | stop exon 38 | 24,349 | 18.0 |
| 4.5 | duplication 8-9 | 18,461 | 20.1 |
| 4.5 | exon 62 splice donor site | 46,964 | 20.8 |
| 4.6 | deletion 45-50 | 10,688 | 11.8 |
| 4.8 | deletion 45-52 | 38,415 | 22.4 |
| 4.9 | deletion 45-50 | 37,595 | 23.3 |
| 5.0 | deletion 3-7 | 16,617 | 14.6 |
| 5.1 | duplication 53-57 | 36,036 | 15.4 |
| 5.1 | nonsense 34 | 33,354 | 21.5 |
| 5.2 | duplcation 14-17 | 34,708 | 24.0 |
| 5.3 | deletion exon 46 | 27,219 | 20.3 |
| 5.3 | deletion 6-19 | 13,230 | 21.1 |
| 5.5 | deletion 45-54 | 30,605 | 19.3 |
| 5.6 | deletion 45-50 | 15,648 | 17.2 |
| 6.1 | deletion 46-49 | 12,625 | 18.2 |
| 6.3 | deletion 51-55 | 36,200 | 20.6 |
| 6.5 | deletion 8-33 | 21,145 | 19.9 |
| 6.7 | deletion 51-54 | 15,626 | 14.2 |
| 6.9 | deletion 49-50 | 23,477 | 21.9 |
| 6.9 | deletion 3-7 | 14,040 | 12.1 |
| 7.5 | deletion 45-54 | 24,791 | 15.9 |
| 7.5 | stop exon 15 | 10,543 | 11.4 |
| 7.6 | duplication 5-7 | 18,334 | 17.7 |
| 7.7 | IVS 12+1 | 9,212 | 16.3 |
| 8.0 | duplication 13-21 | 15,941 | 21.5 |
| 9.7 | deletion 48-50 | 13,273 | 14.8 |
| 11.7 | deletion exon 63 | 1,445 | 4.5 |
| 12.1 | deletion exon 52 | 17,500 | 13.4 |
| 13.8 | deletion 45-50 | 2,444 | 6.7 |
| 14.7 | deletion 46-51 | 1,430 | 3.9 |
| 14.9 | deletion 61-79 | 404 | 1.4 |
| 15.0 | deletion 45-52 | 2,422 | 4.4 |
| 15.6 | deletion 45-50 | 1,353 | 4.2 |
| 15.7 | deletion exon 1 | 455 | 0.9 |
| 15.9 | deletion exon 53 | 1,277 | 2.3 |
| 16.3 | nonsense exon 8 | 434 | 3.3 |
| 16.4 | stop exon 41 | 2,349 | 4.8 |
| 17.5 | duplication46-52 | 732 | 2.0 |
| 17.6 | pre stop exon 30 | 1,416 | 3.5 |
| 18.4 | deletion 46-51 | 876 | 2.0 |
| 19.7 | deletion 49-50 | 356 | 0.7 |
| 19.9 | deletion 50 | 941 | 2.4 |
| 20.0 | deletion exon 62 | 2,347 | 2.5 |


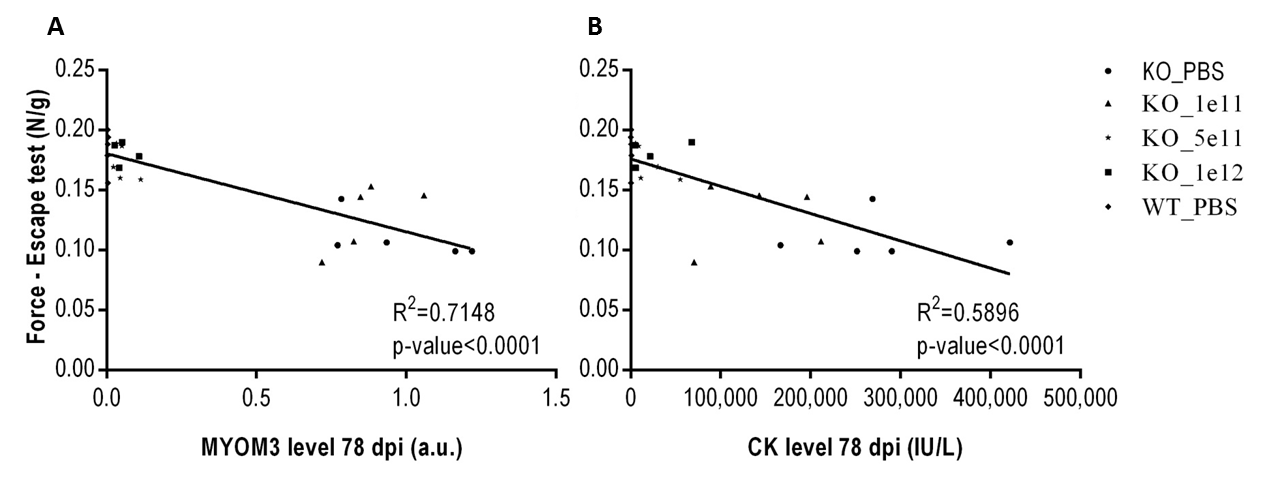


**Figure S1**: Correlation between the levels of the MYOM3 fragments (**A**) or CK-M (**B**) in serum with muscle force evaluated by the escape test. KO_PBS, KO_1e11, KO_5e11, KO_1e12: KO-Sgca mice injected with PBS or the respective dose of the vector. WT_PBS: C57BL/6J control mice injected with PBS. Levels of the MYOM3 fragments and CK-M were estimated by Western blot analysis 78 days post-injection. Dpi: days post-injection.


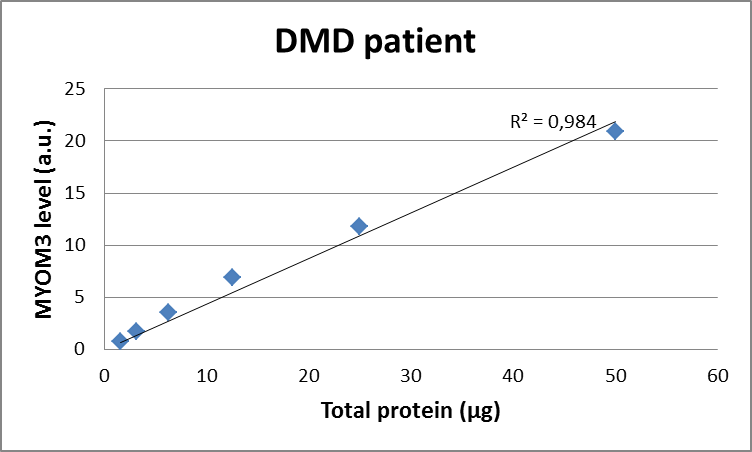

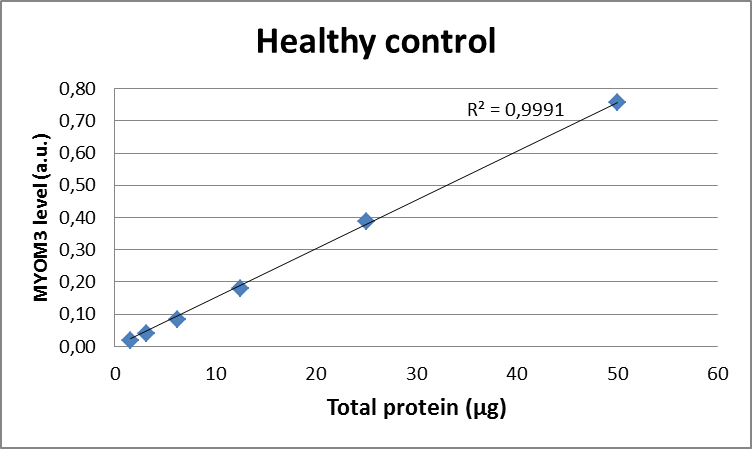


**Figure S2**. MYOM3 Western signal as a function of MYOM3 protein load. Increasing quantities of serum (total serum proteins, from 1.5 µg to 50 µg) from a DMD patient with the highest MYOM3 level (left panel) and from a healthy donor (right panel) were analyzed by Western blot as described in Materials and Methods. In both cases Western blot signals and loaded quantities of MYOM3 were linearly dependent, thereby allowing for MYOM3 quantification over a wide linear dynamic range.

**
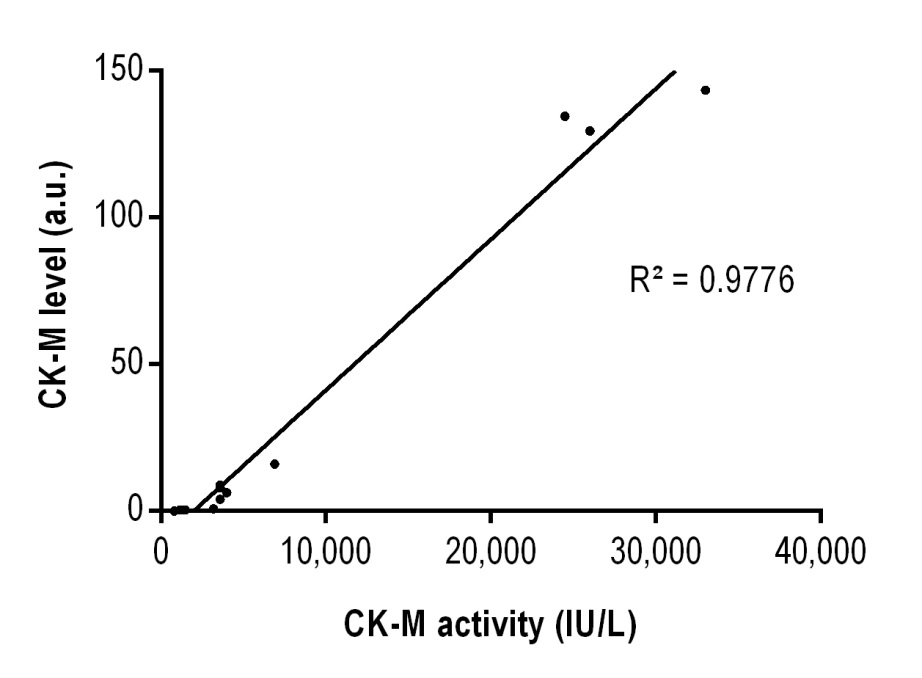
**

**Figure S3.** Correlation between the CK-M measured by Western blot (a.u) and its enzymatic activity (IU/L). Level of CK was measured by two methods in serum of *mdx* mice before and at different time after physical exercises. Three mice and four time points were used to build the graph.


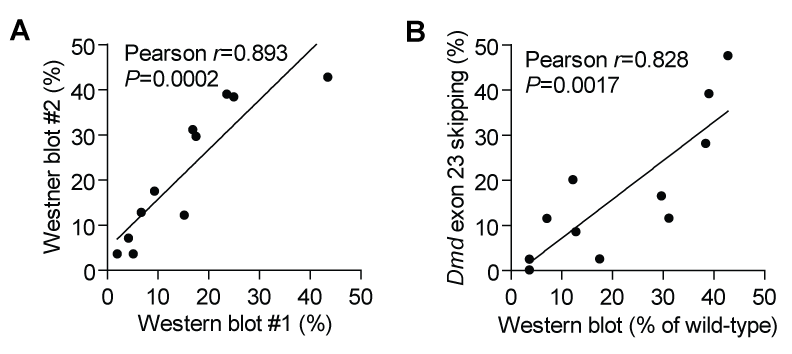


**Figure S4. Validation of dystrophin quantitation methodology.**

To further demonstrate the robustness of this method, we have performed the complete western blot protocol twice on the same samples reported in this study (**Figure A**). Calculated dystrophin expression values were highly positively correlated between the two blots (*r*=0.893, *P*=0.0002) thereby demonstrating the reproducibility of our dystrophin protein measurements. Similarly, dystrophin quantitation by western blot was compared with the levels of *Dmd* exon 23 skipping as determined by RT-qPCR for each sample used in this study (**Figure B**). These completely different techniques also produced data which were strongly positively correlated (*r*=0.828, *P*=0.0017). This independent measure supports the validity of our dystrophin protein measure, at least with respect to comparisons between animals.

To determine if the sample sizes used in this study are sufficient to determine significant differences in the dystrophin quantitation data we performed a power calculation. (We have used a conservative two-tailed setting and an alpha level of 5%). Comparing the 4 week vs 2 week data with *n*=4, we calculate statistical power at 90.8%. Comparing the 8 week vs 2 week data with *n*=4, we calculate statistical power at 100%. These results suggest that the study is sufficiently powered to detect meaningful differences and that assertions to the contrary are not supported by the data.
